# An Alarming Sign: Acute Limb Ischemia in Patients With COVID-19

**DOI:** 10.7759/cureus.80798

**Published:** 2025-03-18

**Authors:** Mohd Faris B Mohd Aladin, Rosenelifaizur Ramely

**Affiliations:** 1 General Surgery, Raja Perempuan Zainab II Hospital, Kota Bharu, MYS; 2 Surgery, School of Medical Sciences, Universiti Sains Malaysia, Kota Bharu, MYS

**Keywords:** acute limb ischaemia, covid-19 acute limb ischemia, covid-19 pandemic, fogarty embolectomy, hypercoagulable state, thromboembolic disease

## Abstract

Acute limb ischemia (ALI) can occur as a late complication in patients recovering from COVID-19. Although typically seen during the acute phase, ALI may present weeks to months later, making diagnosis more challenging. Symptoms, including acute limb pain, pallor, coldness, and absent pulses, can be misattributed to other post-viral issues. COVID-19 induces a prothrombotic state that persists after recovery, with increased D-dimer, fibrinogen, and endothelial dysfunction contributing to thrombus formation. Early diagnosis through clinical assessment and imaging is crucial to prevent irreversible limb damage. Treatment involves anticoagulation, revascularization, and supportive care, tailored to the patient's condition. Vigilance for late-onset ALI in post-COVID-19 patients is essential to improve outcomes and prevent limb loss.

## Introduction

In February 2020, Malaysia reported its first confirmed case of coronavirus disease 2019 (COVID-19) in Kuala Lumpur. While respiratory complications such as viral pneumonia and acute respiratory distress syndrome (ARDS) are the most recognized manifestations of COVID-19, increasing evidence suggests that the virus also induces a hypercoagulable state, predisposing patients to thrombotic complications [1].

This prothrombotic state results from endothelial dysfunction, systemic inflammation, and excessive activation of the coagulation cascade, leading to an increased risk of conditions such as deep vein thrombosis, pulmonary embolism, and acute limb ischemia (ALI) [2]. ALI, characterized by a sudden reduction in blood flow to a limb, is a vascular emergency that can lead to severe morbidity, including limb loss, if not promptly diagnosed and treated.

Given the growing recognition of ALI as a life-threatening complication in COVID-19 patients, maintaining a high index of suspicion is crucial. Early identification and timely intervention, including anticoagulation or revascularization, are essential to improving patient outcomes and preventing irreversible ischemic damage.

## Case presentation

A 67-year-old female, recently discharged from the hospital for COVID-19 infection (category 4, with pneumonia requiring oxygen therapy) on day 16 of illness, complained of sudden right leg pain starting at 4 pm the same day. She had no prior limb pain, with sudden onset occurring this evening. Due to the pain, she sought her general practitioner's care and was prescribed analgesia. However, her symptoms did not resolve.

Her past medical history was not relevant; there was no previous medical history of atrial fibrillation, venous thromboembolism, cardiovascular disease, or cerebrovascular disease. Furthermore, she did not have severe respiratory symptoms and stayed home and isolated herself from others. She decided to come to the emergency department because of severe pain and reduced sensation in the lower limb, which had become progressively worsening.

The patient’s right leg demonstrated prominent coldness and pallor, with sensory deficits in the right lower limb and thigh. There were no abnormal motor or neurological findings in either right thigh or lower limb. The right popliteal (PA), posterior tibial artery (PTA), and dorsalis pedis artery (DPA) pulses were absent, but the right femoral artery pulses were detectable, 2+. There was no Doppler signal in either the right PA, PTA, or DPA. The patient's diagnosis was acute limb ischemia Rutherford class 2b. Table 1 presents the results of the first laboratory study.

**Table 1:** Initial laboratory investigation. F: female

Variables	Result	Unit	Normal range
White blood cells (WBC)	17.6	x10^9^/L	F: 3.40-10.1
Hemoglobin (Hb)	15	g/dL	F: 11.6-15.1
Platelet	98	x10^9^/L	F: 158-410
D-dimer	70.1	µg/mL	<0.45
Creatinine kinase (CK)	472	U/L	26-192
C-reactive protein	153	mg/L	<5

The examination of the contralateral leg was unremarkable. A physical examination demonstrated a blood pressure of 156/72 mmHg, a heart rate of 65 beats per minute, and a temperature of 36.4°C. Her respiratory rate was 22 breaths per minute, and her oxygen saturation was 98% under room air. Lung and abdomen examinations were unremarkable.

**Figure 1:**
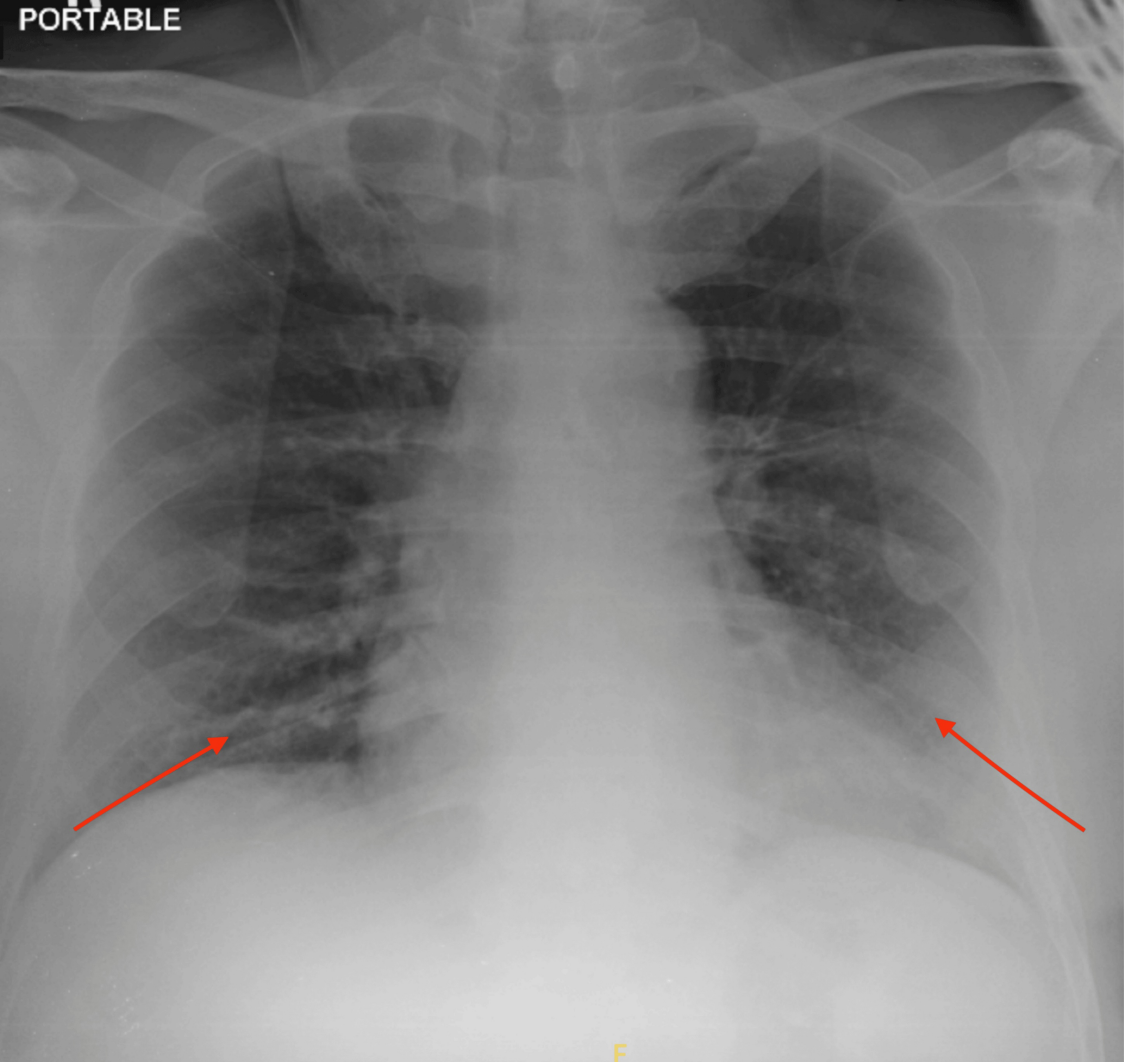
Increased reticular opacities at both lower zones (arrows) suggestive of lung infection.

**Figure 2:**
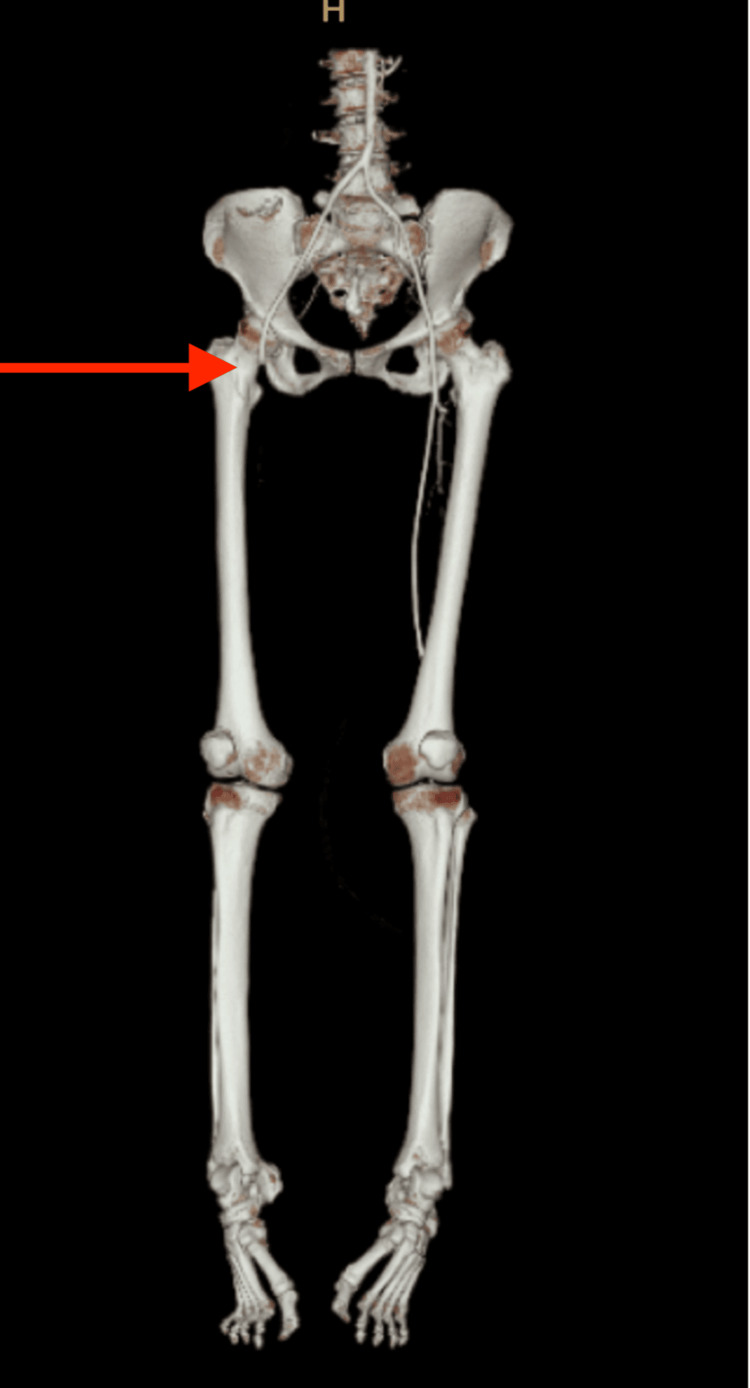
Long segment of acute thrombosis of right lower limb arteries involving right common femoral artery until distally (arrow).

A chest X-ray demonstrated increased reticular opacities at both lower zones, which is typical of COVID-19 pneumonia (Figure 1). She underwent computed tomography (CT) angiography, which demonstrated long-segment acute thrombosis of the right lower limb arteries involving the right common femoral artery until distally (Figure 2). As a result, an emergency embolectomy was planned, and she was taken to the operating room for surgery.

We performed an embolectomy through the right common femoral artery. An arteriotomy was made, revealing an occlusive embolus within the vessel. A long, gelatinous clot was extracted. The clot was removed proximally from the right common femoral artery and fragmented using 4Fr Fogarty catheters, which improved back bleeding. The embolectomy was then extended distally to the profunda and superficial femoral arteries using 3Fr and 2Fr Fogarty catheters. The effectiveness of restored blood flow was assessed intraoperatively using Doppler evaluation to confirm vessel patency and adequate perfusion to the distal arteries.

After surgery, the patient was transferred to the general ward, and intravenous heparin infusions were started. She showed a good recovery post-operatively and was discharged after five days of admission with an oral medication regimen of warfarin 5 mg once daily with a target international normalized ratio (INR) of 2-3. After a follow-up after 14 days of embolectomy, she had no complications and was able to walk without pain.

## Discussion

This case report presents a confirmed case of COVID-19 complicated by acute limb ischemia (ALI), further contributing to the expanding body of evidence that suggests a significant association between COVID-19 and the development of incident arterial thrombosis. As the COVID-19 pandemic has unfolded, clinicians have increasingly recognized that the virus can affect not only the respiratory system but also induce systemic thrombotic events, including ALI. This study adds to the growing understanding that COVID-19 patients, even those without prior thrombotic risk factors, may develop ALI, an acute condition often linked to severe vascular events [3].

This aligns with previous reports, including a study by Santosa and Yuwono, who observed that COVID-19 patients who developed ALI were often without prior thrombotic conditions. Their research also underscored that ALI can occur later in the course of the disease, even after the acute viral symptoms have subsided or stabilized. The authors suggested that this could be due to a prolonged hyperinflammatory state and a delayed but significant increase in clotting risk, which contributes to the development of thrombotic events like ALI in the later stages of hospitalization [4].

Our patient developed ALI 16 days after being diagnosed with COVID-19. This delayed onset of ALI is significant and aligns with findings from Santosa and Yuwono, who emphasized that the temporal relationship between COVID-19 and ALI could span the entire hospitalization period. This delay is notable because it indicates that ALI may not only be a consequence of the initial acute phase of COVID-19 but could also emerge as a complication in patients as the disease progresses, particularly in severe cases. COVID-19 has been shown to induce an intense inflammatory response characterized by a cytokine storm, which may persist and increase the risk of thrombosis even after the initial viral replication phase has resolved [4].

The pathophysiology underlying these thrombotic events is multifactorial. COVID-19 causes endothelial injury through both direct viral infection of endothelial cells and an indirect effect via the body's immune response. This injury leads to the release of prothrombotic factors and a disruption in the balance between pro- and anti-coagulant factors. The virus can also trigger a hypercoagulable state by stimulating the production of cytokines, such as interleukin-6 (IL-6) and tumor necrosis factor-alpha (TNF-α), which promote coagulation. Additionally, the virus can lead to an increase in fibrinogen levels, platelet activation, and increased clotting factor production, which all contribute to an elevated risk of thrombus formation. This hypercoagulability is compounded by the proinflammatory environment created by the viral infection, which not only disrupts normal hemostasis but also enhances the formation of blood clots, particularly in the microvascular and macrovascular systems [5].

Given the association between COVID-19 and thrombotic complications, including ALI, it is crucial for clinicians to remain vigilant about the risk of hypercoagulability. One of the most useful biomarkers in this regard is D-dimer, a degradation product of fibrin that is released when blood clots are broken down. Elevated D-dimer levels are a hallmark of a hypercoagulable state and can serve as an early indicator of thrombotic complications in COVID-19 patients. A rise in D-dimer levels is particularly concerning because it signifies ongoing clot formation and increased risk for complications like ALI, DVT, and PE. Several studies, including one by Zhang et al., have shown that elevated D-dimer levels correlate with worse outcomes in COVID-19 patients, including a higher incidence of thrombotic events, mortality, and organ failure [6].

## Conclusions

In conclusion, this case reinforces the importance of considering ALI as a potential complication in COVID-19 patients, particularly those who experience severe disease and prolonged hospitalization. It also emphasizes the need for vigilant monitoring of thrombotic markers, especially D-dimer, to identify patients at higher risk for thrombotic complications. Given the complex pathophysiology of COVID-19 and its ability to induce a hypercoagulable state, healthcare providers must remain attentive to a wide spectrum of vascular complications, ensuring timely interventions to mitigate these risks.
